# Proposal and Strategy for Nursing-Led Research: Protocol for an Unfunded Clinical Trial

**DOI:** 10.2196/56062

**Published:** 2025-02-10

**Authors:** Leticia Carmen Simón-López, Ismael Ortuño-Soriano, Raquel Luengo-González, Paloma Posada-Moreno, Ignacio Zaragoza-García, Rubén Sánchez-Gómez

**Affiliations:** 1 Support Unit of the General Directorate of Public Health and Equity in Health Ministry of Health Madrid Spain; 2 Nursing Department Universidad Complutense de Madrid Madrid Spain; 3 IdISSC Instituto de Investigación Sanitaria del Hospital Clínico San Carlos Madrid Spain; 4 Nursing and Physiotherapy Department Universidad de Alcalá Alcalá de Henares Spain; 5 I+12 Research Institute Hospital 12 de Octubre Madrid Spain

**Keywords:** clinical trial, academic trial, nonfunded, commercial, nurse-led, low intervention, health product, peripheral venous cannulation, PVC, protocol, randomized controlled trial, RCT, adults, healthy adults, funding, academic sponsors, cause-effect results, insurance

## Abstract

**Background:**

Clinical trials are known to provide cause-and-effect results and data with low levels of bias. However, a lack of funding for clinical trials, which are considered expensive, means that academic sponsors are rarely able to conduct them. Academic trials are considered highly relevant for the valuable results they provide for clinical questions. This is why initiatives to conduct unfunded clinical trials have been identified as an important issue to pay attention to in future studies. Therefore, we present our initiative through Rogers’ theory, which is highlighted in the literature for diffusing innovative change across organizations.

**Objective:**

The purpose of this paper was to describe our case regarding management for conducting a nonfunded nurse-led clinical trial based on our previous low-interventional clinical trial across a specific health organization and with nurses.

**Methods:**

We conducted a low-intervention, nonexternally funded clinical trial using the human and material resources available on site. We managed our trial in a clinical trial unit where there were staff, sources, and ongoing commercial clinical trials. We conducted our trial based on an ongoing commercial trial, and, to do so, we needed behavioral changes. We relied on Rogers’ theory, and we identified strengths and barriers to change by analyzing actors' characteristics, perceptions of the situation, motivation, and information. Afterward, we divided the staff according to their characteristics related to innovation and change into permanent staff (research staff with a culture of change) and nonpermanent staff (nursing staff with occasional attendance and resistance to change). First, we preselected only those nurses who were more aware of change (innovators and pioneers) to participate in our trial to avoid a massive rejection, and later, we asked others to join (late adopters). We followed Rogers’ phases. For research staff who were aware of the funding, we focused on the “persuasion phase,” while for nursing staff, we mixed the “knowledge and persuasion phases” and used pioneers and early adopters as a positive example for other nurses as well as nonfinancial incentives (persuasion). Our trial consisted of different methods of vein cannulation, which was performed in the ongoing commercial trial. Thus, the entire development of our low-interventional clinical trial was conducted without interfering at any point with the parallel commercial clinical trial.

**Results:**

Our management allowed effective conduct of our study, and we met our aims without external funding and without ethical impact during the commercial clinical trial. Costs remained low, primarily because the major expenses were covered by the commercial clinical trial as an inherent part of its design.

**Conclusions:**

Our initiative to conduct a low-intervention clinical trial with no or limited funding was cost-effective. This initiative can be used by researchers with valuable academic research questions who do not have the external funding to conduct studies.

**Trial Registration:**

ClinicalTrials.gov NCT04027218; https://clinicaltrials.gov/study/NCT04027218

**International Registered Report Identifier (IRRID):**

RR1-10.2196/56062

## Introduction

### Background

Clinical trials are known to provide cause-and-effect results and usually yield high-quality data due to low levels of bias [1]. In addition, clinical trials not only provide the best context for advancing clinical research and health care but also create opportunities to reduce health care costs [2]. Moreover, clinical trials involve key personnel, such as nurses, who can be recruited and trained to carry out a protocol [3]. In the case of clinical research nurses, their ability to work independently has been recognized, but they face practical, ethical, and resource challenges [4]. In particular, the lack of funding for clinical trials, which are considered expensive and involve additional effort (especially investigator-led clinical trials), leads to voluntary activities [5]. It should also be noted that academic sponsors are rarely able to conduct trials without external funding [6]. Therefore, it has become clear that there is a need for increased coordination to ensure that robust research is conducted and to adopt adaptive trial design strategies to respond to the rapidly evolving evidence landscape [7].

Nevertheless, academic trials (ie, trials in which the comparator is usually standard clinical practice [1]) are considered highly relevant due to the valuable results they provide when investigating clinical questions [8]. Academic trials enable comparative data and may lead to changes in practice [5], but researchers in such trials face multilevel challenges, most notably applying for grants and managing funds to pay for participation or study-related injuries [9].

In Europe, between 10% and 30% of clinical trials are conducted by academic or noncommercial sponsors [8], and only a limited number of nurses receive support through funding mechanisms [10] despite evidence showing that trials led by a nurse or physician have noninferior results [11].

Therefore, although our trial is registered at ClinicalTrials.gov (NCT04027218) and our results are published [12], initiatives to conduct nonfunded clinical trials, such as low-interventional clinical trials, have been identified as a major issue to be targeted in future studies [8]. However, implementing initiatives, new ideas, or innovative processes across an organization is challenging. The literature suggests the use of Rogers’ curve or Rogers’ diffusion of innovation theory to introduce innovation because it applies within and across organizations [13], such as in schools and for teachers [14,15] as well as in health care organizations [16].

### Objective

The purpose of this paper was to describe our case of managing the conduct of a nonfunded, nurse-led clinical trial across a specific health organization and with nurses.

## Methods

### The Field and Study Context

The aim of our initiative was to conduct a phase 4 low-interventional clinical trial without external funding that resulted in low bias, was of high quality, and used available onsite human and material resources.

Thus, we managed our trial in a clinical trial unit where staff and sources were already available. However, up until our trial, almost all clinical trials in this unit evaluated drugs, were financed by the pharmaceutical industry, and involved procedures, particularly venipuncture by nurses, that were performed in the same way.

In other words, the nurses and other research staff in the unit did not participate in a culture of conducting different clinical trials, such as our clinical trial involving medical devices and that proposed changing “the assembly line.” In our case, a clinical trial was conducted with different forms of venipuncture, which had previously always been performed using the same technique. This involved changing certain positioning and behavioral barriers.

In the clinical trials unit, there were fixed staff (researchers) and variable staff (nurses). The variable staff consisted of a pool of 20 to 25 nurses who came on occasional days to support the clinical trials but whose main activity was in other hospital departments.

### Management and Sampling Access

Rogers’ 5 stages of innovation decision-making consist of knowledge, persuasion, decision, implementation, and confirmation information [16]. There are different roles to implementing change. His theory shows that the following roles exist in certain proportions: innovators (2.5%), early adopters (13.5%), adopters (34%), late adopters (34%), and nonadopters (16%). He also considered the minimum threshold for change to be the sum of innovators and early adopters.

Thus, we contemplated the “knowledge phase” for the fixed and variable staff. In the case of workers in the fixed staff component, they were already aware of the problem of obtaining funding to pay employees, so we only focused on the “persuasion phase” as we aimed on getting approval from the director of the unit, who introduced our clinical trial to the fixed and variable staff members.

Another exception had to be made at this stage regarding the expert in blood sample analysis (research staff), as we needed him to analyze our indicators in addition to those that were requested for the unit’s trials. Persuasion consisted of suggesting authorship in publications given his interest in his scientific career and the potential for indirect income.

For the variable staff (nurses), we mixed the “knowledge and persuasion phases.” We chose to invite only nurses who had the most knowledge or awareness of the change (knowledge) to participate in our clinical trial. In other words, we preselected the nurses (innovators and early adopters) to avoid mass rejection of the trial and to try to allow other nurses to see that a few nurses (innovators and early adopters) had seen this change as a positive step (persuasion). We did not conduct a survey to classify the nurses according to Rogers’ roles because we knew the pool of our nurses well and we already knew their positions.

Specifically, to carry out the research, the nurse in charge of this study could not offer any direct payment. We offered to thank the staff in publications, and if anyone wanted to play a more active role in writing manuscripts about this study, they would be positively considered for authorship of articles resulting from the study. The publications are considered professional merit in our country and serve to increase the salary in one’s professional career.

Nurse innovators and early adopters agreed to appear in acknowledgements (they did not want to have a more active role) and contribute to the culture and advancement of innovation and research in the nursing profession for colleagues who did not have this insight (late adopters mainly).

In the implementation phase, some nurses (adopters) asked the nurse in charge of the study about the new technique being performed by some collaborating nurses in this study. Therefore, we took advantage of this interest to invite more than one-half of the pool of nurses (adopters and late adopters) to participate in our study. We had a small group of nurses who we knew would not be interested in participating (nonadopters), and they were the last to be invited to participate and declined.

In relation to the fixed staff (research staff), we only focused on the “getting to know” and “confirmation” phases, as they already had knowledge of the project. They were observers, and we only gave them a 20- to 30-minute training on how the study was going to be developed in the unit at the same time a phase 1 clinical trial was being conducted (which was the routine work of the unit) and sought their confirmation to be aligned with the project.

### Trial Design

Although our clinical trial protocol was already registered at ClinicalTrials.gov and is freely accessible, we deemed it necessary to provide a brief summary of our trial design so that our management of the field and study context were fully understood.

### Study Sample and Eligibility Criteria

Our participants were recruited from the population of individuals who provided written informed consent for the primary clinical trial at the unit (phase 1 bioequivalence study). As shown in Table 1, on the night of first admission (visit 2 in phase 1), participants in the phase 1 bioequivalence study were invited to enroll in our phase 4 clinical trial. They signed the informed consent at that time. Participation in our phase 4 clinical trial was voluntary. We informed participants that this study was a nonfunded study and that no incentive would be provided beyond the payment they received from the phase 1 trial. We also communicated that the potential benefit for them was the expected effective interventions hypothesized in our phase 4 trial (Table 2).

The inclusion and exclusion criteria were the same as those for the bioequivalence trial. In addition, we added 3 criteria for our phase 4 clinical trial that were also compatible with the criteria for the phase 1 trial. These 3 criteria were 6 hours to 8 hours of fasting before vein cannulation, fluid intake limited to ≤500 mL 6 hours to 8 hours before venous cannulation [17], and having been a former participant in a bioequivalence clinical trial at our hospital.

**Table 1:** Procedures of the phase 1 clinical trial.

Procedures	Visits												
	0	1. Screening^a^	2. Day 1 of first entry^b^	3. Day 2 of first entry^c^	4. Day 3 of first entry^d^	5. Day 4 of first entry^e^	6. Day 4 of first entry^f^	Washout^g^	7. Day 1 of second entry^h^	8. Day 2 of second entry^i^	9. Day 3 of second entry^j^	10. Day 4 of first entry^k^	11. Day 4 of first entry^l^
Informed consent	✓	—^m^	For the phase 4 trial	—	—	—	—	—	—	—	—	—	—
Inclusion and exclusion	✓	✓	✓	✓	—	—	—	—	—	—	✓	✓	—
Concomitant medications	—	✓	✓	✓	✓	✓	✓	—	✓	✓	✓	✓	✓
Blood and urine analysis	—	✓	—	—	—	—	—	—	—	—	—	—	—
Medical history	—	✓	—	—	—	—	—	—	—	—	—	—	—
Physical examination	—	✓	—	✓	—	—	—	—	—	✓	—	—	—
Height	—	✓	—	—	—	—	—	—	—	—	—	—	—
Weight	—	✓	—	✓	—	—	—	—	—	✓	—	—	—
Electrocardiogram	—	✓	—	✓	—	—	—	—	—	✓	—	—	—
Vital signs (HR^n^, BP^o^)	—	✓	—	✓	—	—	—	—	—	✓	—	—	—
Tympanic temperature	—	✓	—	✓	—	—	—	—	—	✓	—	—	—
Peripheral vein catheterization	—	—	—	✓	—	—	—	—	—	✓	—	—	—
Pharmacokinetic blood basal sample	—	—	—	✓	—	—	—	—	—	✓	—	—	—
Drug administration	—	—	—	✓	—	—	—	—	—	✓	—	—	—
Pharmacokinetic blood samples	—	—	—	✓	✓	✓	✓	—	—	✓	✓	✓	✓
Venepuncture	—	—	—	—	✓	✓	✓	—	—	—	✓	✓	✓
Adverse events record	—	—	✓	✓	✓	✓	✓	—	✓	✓	✓	✓	✓

^a^Up to 3 days after visit 0.

^b^1 week after screening.

^c^10 hours after visit 2.

^d^24 hours after visit 3.

^e^48 hours after visit 4.

^f^72 hours after visit 5.

^g^1 week after visit 6.

^h^Up to 24 hours after washout.

^i^10 hours after visit 7.

^j^24 hours after visit 8.

^k^48 hours after visit 9.

^l^72 hours after visit 10.

^m^Not applicable.

^n^HR: heart rate.

^o^BP: blood pressure.

**Table 2:** Procedures of the phase 4 clinical trial.

Procedures	Visits										
	0	1	2	3	4	Washout	5	6	7	8	9
Informed consent	✓	—^a^	—	—	—	—	—	—	—	—	—
Inclusion and exclusion	—	✓	—	—	—	—	—	✓	—	—	—
Vein perception	—	✓	—	—	—	—	—	✓	—	—	—
Sequence allocation	✓	—	—	—	—	—	—	—	—	—	—
Intervention or comparator	—	✓	—	—	—	—	—	✓	—	—	—
Pain assessment	—	✓	—	—	—	—	—	✓	—	—	—
Hemolysis	—	✓	—	—	—	—	—	✓	—	—	—
Skin type assessment	—	✓	—	—	—	—	—	—	—	—	—

^a^Not applicable.

### Randomization, Allocation, and Sample Size

Participants were randomized to 1 of 3 interventions and one of the sequences of applying those interventions within 2 periods. Thus, we allocated sequences of 1 intervention and a comparator (1 sequence for each participant).

**Figure 1:**
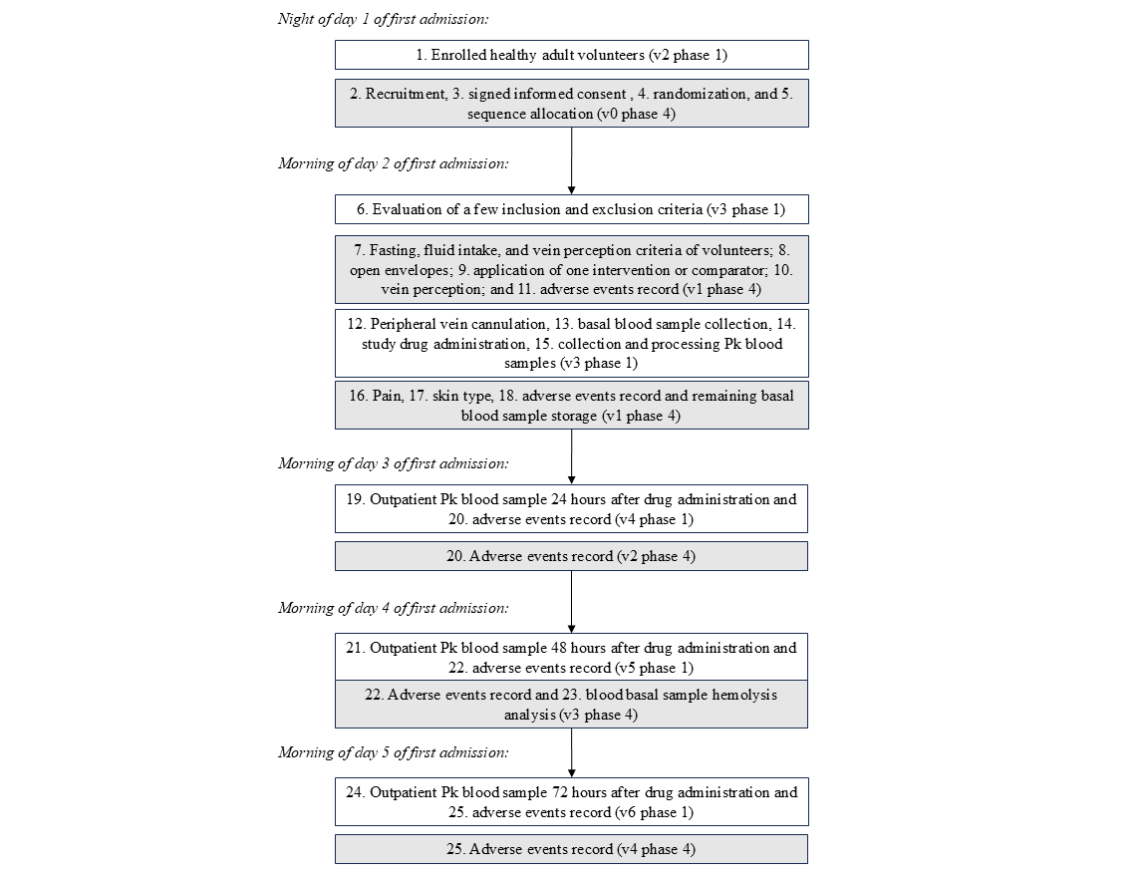
Combined fieldwork of the phase 1 and 4 clinical trials. Pk: pharmacokinetics; v: visit.

As Figure 1 shows, randomization was performed at visit 0 of the phase 1 clinical trial after informed consent form was obtained and before participants were screened for inclusion and exclusion criteria for both the phase 4 and 1 trials. As shown in Table 3, we designed 6 sequences.

The study was carried out in the clinical trial unit at our hospital, where bioequivalence clinical trials (phase 1) were performed with groups of 12 participants. Accordingly, we duplicated the 6 sequences used for each group of participants.

**Table 3:** Description of the sequences.

No	Sequence	First period	Second period
1	Dry topical heat, comparator	Dry topical heat	Comparator
2	High pressure, comparator	High pressure	Comparator
3	Dry topical heat and high pressure, comparator	Dry topical heat and high pressure	Comparator
4	Comparator, dry topical heat	Comparator	Dry topical heat
5	Comparator, high pressure	Comparator	High pressure
6	Comparator, dry topical heat and high pressure	Comparator	Dry topical heat and high pressure

### Interventions and the Comparator

Our interventions, given that we designed the additional eligibility criteria, were compatible with the interventions planned in the bioequivalence clinical trial. In the bioequivalence clinical trial, venous cannulation is always performed to obtain blood samples, and we established the following interventions and comparator to avoid interfering with those in the phase 1 trial. First, to apply dry topical heat, 2 bags were heated and placed on each participant’s forearm for 7 minutes while an elastic compressor was applied [18]. Second, high pressure was applied via a sphygmomanometer cuff set at 100 mm Hg [19]. Third, for the combined intervention, dry heat was applied followed by pressure per interventions 1 and 2. The 3 interventions had a common comparator (ie, the elastic compressor), which was provided by the hospital and used according to CLSI GP41-A6 guidelines [20], as was performed in the phase 1 clinical trial.

### Measures

Vein perception was assessed using the Venous International Assessment scale, which is a validated scale [21]. Vein cannulation was performed using a 20-gauge diameter catheter, and an EDTA K2 blood sample was collected using a Vacutainer blood collection tube (Becton, Dickinson and Company). Pain was assessed using the visual analogue scale, which is validated for acute pain [22]. Skin type was assessed using the Fitzpatrick scale, which has been previously validated [23]. Adverse events were assessed with a severe causality algorithm from the World Health Organization (WHO) [24].

The aforementioned materials are routinely used by nurses in the clinical trial unit and in clinical practice at our hospital. The nurses were familiar with the scales except for the Venous International Assessment scale, Fitzpatrick scale, and WHO algorithm. Therefore, collaborative nurses were trained to administer these tools. We also measured hemolysis in our blood samples, which was performed by an expert who routinely used a NanoDrop 2000 Spectrophotometer (ThermoFisher Scientific) at the unit. Again, we used human and material resources already available in the unit.

### Adherence and Monitoring

Our phase 4 clinical trial involved the use of nonharmful interventions. Our interventions delayed vein cannulation by only 7 minutes (heat application), and the data collection did not require extra visits or blood draws. Because our phase 4 clinical trial was a nonfunded study, economic incentives for participants came only from the phase 1 commercial clinical trial. Therefore, the phase 1 trial guaranteed a low dropout rate and adherence to our clinical trial.

According to the low-interventional clinical trial regulations [25]*,* an external monitor was not provided, and the nurse principal investigator conducted the study.

### Ethical and Financial Considerations

The research protocol and methodology were approved by the Ethics Committee of the Hospital de La Princesa (Madrid, Spain) under code ECYPVEN-H/17 and registration number 3113.

This was considered a low-interventional clinical trial because “the intervention poses only very limited additional risk to the subject compared to normal clinical practice.” We performed our clinical trial based on a phase 1 clinical trial that involved a vein cannulation procedure to ensure participant safety in relation to the same procedure in other clinical trials. Thus, we did not require specific insurance for any potential injury to participants; they were covered by the phase 1 insurance or health system insurance. Data from participants in the phase 1 clinical trial were coded for our study to ensure privacy [25].

Both clinical trial protocols and informed consent forms were in accordance with the Declaration of Helsinki [26]. Specifically, as our clinical trial was concerned with nursing, announcements and information about this trial were made by the nurse who was the principal investigator.

The participants did not receive any remuneration for participation in the low-intervention trial or any other type of compensation.

Furthermore, none of the information appearing in this article allows the identification of data or images of the participants.

We calculated that less than €200 (US $209.50) would be sufficient to cover the overall costs of our phase 4 study, including materials required for our interventions and comparators: 6 pairs of carob seed bags for heat application (4 pairs for use and 2 pairs for backup), 5 sphygmomanometers for pressure application (4 for use and 1 for backup), and 4 timers.

The aforementioned cost overrun was mainly for materials, as the rest of the costs were covered because we used resources from the clinical trials unit where the phase 1 study was conducted.

### Blinding

This open study was justified by the complexity of masking interventions (heat or pressure) and operators, as proposed by the CONSORT (Consolidated Standards of Reporting Trials) Statement for Randomized Trials of Nonpharmacologic Treatments [27]. Only the biologist who analyzed plasma sample absorbance was blinded [27].

## Results

Our management was effective for conducting a low-intervention study, and we met our objectives without external funding. We conducted a clinical trial in the clinical trial unit of Hospital Universitario de La Princesa (Madrid) during the months of June 2017 and July 2017 with 59 healthy adults who were randomly allocated to 1 of 3 interventions: (1) using dry topical heat for 7 minutes produced by 2 hot seed bags (n=21), (2) applying controlled pressure from a sphygmomanometer inflated to 100 mm Hg (n=18), and (3) combining heat and pressure (n=20). We found that the pressure intervention (n=18) was the most effective for relieving pain, followed by heat (n=21) and the combined intervention (n=20). Furthermore, hemolysis was not significantly affected by any of our interventions, and no serious adverse events occurred [12]. None of the participants dropped out of the study, and a total of 10 nurses who had at least 1 year of experience in the clinical trial unit contributed to the fieldwork of the trial.

## Discussion

### Main Findings

Our strategy was effective for carrying out a low-intervention, academic clinical trial, as claimed by previous studies. Therefore, we were able to provide an answer to a problem detected in the scientific literature [8].

Our results [12] were consistent with those of previous parallel clinical trials that were funded by (1) a foundation and involved a specific device provided by a manufacturer [18] and (2) a grant [28].

### Comparison With Prior Work

Similar to our study sample of 59 participants [8], additional studies have been conducted using 68 participants per group [18] and 36 and 34 participants in 2 groups [28]. In contrast, our study was a crossover, nonfunded study [12]. Although a previous study stated that noncommercial clinical trials recruit fewer participants than commercial trials [1], presumably due to the lack of financial compensation, we could not confirm that statement based on our results.

In addition to the clinical benefits, our results supported our strategy and showed how nurses, who receive relatively little funding (39.4% of total National Institutes of Health funding), can benefit from research funding [29] and lead a high-quality clinical trial without funding. Nurses in a variety of positions are involved in clinical trials, including clinical research nurses (69.7%), research nurse coordinators (17.9%), nurse practitioners (4.4%), and clinical and administrative or program support staff (8%) [30], even if they are not principal investigators [3]. Many are involved in oncology clinical trials [30]. In contrast, our study was conducted with a nurse as the principal investigator [12].

Our trial management was in accordance with the standard framework of Core Competency Domains by the Joint Task Force for Clinical Trial Competency, which consists of 8 domains [31]. Specifically, our study met domains 1 (research design) and 5 (study and site management), which describe a cost-effective, low-interventional clinical trial design and a commercial clinical trial design, respectively. Additionally, domain 7 (leadership and professionalism) [31] was met because our principal investigator was a nurse scientist with a PhD, which the literature highly recommends for research management [3,32].

According to the Rogers’ management curve or theory, contextual factors are crucial and, although we were in a suitable environment (clinical trial unit), both the variable and fixed staff had standardized working procedures to reduce variables. Therefore, paradoxically, we agree with other studies [16] that negotiations (persuasion) for the diffusion of innovation are the biggest complication in those environments where there is no routine development of innovative concepts. However, the fixed staff members were easier to persuade due to their professional profile and career and the possibility of authorship in publications or other merits. Furthermore, unlike the aforementioned study, we did not apply the 5 stages of this theory to all variable and fixed professionals, as the latter had a more advanced research and innovation culture.

We also agree with Lundblad [13], as we were able to establish this theory across the health care organization and in a field where work is dedicated to improving research but the traditional theoretical basis does not include diffusion innovation, as in the variable nursing staff. For this reason, we consider our work to be groundbreaking in a collective that is resistant to change [33]; therefore, we could be introducing an innovative initiative according to Rogers’ curve.

We also agree with the previous study that less complex innovations (such as our research procedure of vein cannulation) are adopted more quickly than those where the adopter must develop new skills and understanding [16].

Unlike the previous study, we did not conduct an interview to categorize staff profiles according to Rogers’ theory, because we believed we knew our pool of nurses (variable staff) and research staff (fixed staff) well and did not need to obtain more information for profile categorization.

We consider that we used the theory adequately, as we were able to conduct our clinical trial and conclude with published results. Furthermore, we agree that this theory is very social, and it depends mainly on two important factors: interpersonal communication relationships and similar actors [13,16]. These were nurse to nurse in our study, rather than doctor or employer to nurse. In our study, the nurses in the pool had very strong rapport and even had a WhatsApp group. Because they asked each other questions through the chat group, they were able to diffuse this innovation. As recommended by Afraz et al [16], we used the innovators as a factor for promoting diffusion, and we demonstrated that it was effective.

A total of 36.5% of registered clinical trial protocols are sponsored by universities, hospitals, and other academic and nonprofit agencies worldwide [34], although the rate is lower in Spain (ie, 10%-30%) [8]. Noncommercial registered protocols are mainly for phase 4 studies and unmasked, controlled clinical trials. Additionally, only 39.4% of noncommercial protocols registered in ClinicalTrials.gov were published in peer-reviewed scientific journals [8]. However, our nonfunded study was an open phase 4 clinical trial registered in a database and published in a peer-reviewed scientific journal [12].

Additionally, we believe that our nonfunded management benefited from industry-sponsored clinical trials in study design, site selection, quality recruitment support, clinical coordinator centers, and access to study databanks, as Laterre and François [35] proposed that academic and industry trials should be constructive and not opposed.

Controversial statements declare that, compared with commercial studies, academic clinical trials are less restrictive with regards to inclusion and exclusion criteria, have less complex protocols, and have higher external validity than internal validity [1]. Others have reported that methodology clinical trials are as valid as commercial and noncommercial clinical trials; however, blinded and multicontinental trials that are usually conducted by major pharmaceutical companies are still considered superior [34]. Conversely, we believe that academic trials could be as restrictive as commercial trials if they are designed like commercial trials, given that our results were in line with such funded studies [18,28].

Our findings also agree with those of Fuentes Camps et al [8], who highlighted the scarcity of economic resources and suggested that initiatives such as low-interventional clinical trials could fill the void. Our low-interventional management optimizes scientific research by conducting a clinical trial at a cost of approximately €153 (US $186) and without ethical concerns or injury to the participants of the commercial clinical trial.

Clinical trials require specific insurance to cover the potential risks; thus, funding is required [36]. However, clinical trials funded by grants, public institutions [5], or associations [36] usually do not have all their costs met [36]. Therefore, our strategy for a low-interventional trial could be a solution when little or no funding is available. Commercial clinical trials could assume 15% to 22% and 11% to 29% of total costs for clinical procedures and administrative concerns, respectively [5].

Additionally, project management was identified as having a high impact on the total costs of a clinical trial [5] and is usually performed by a coordinator [26]. We agree with the proposal by Bevans et al [30] that a principal investigator who coordinates a single-center clinical trial reduces costs without assuming extra effort.

Contrary to our management, a previous study proposed that a better choice to decrease research costs would be to add a hospital employee to the research team instead of modifying the study design [37]. Although the research question guides the study design [37], we consider that, sometimes, a modified study design could contribute superior benefits from a nonfunded clinical trial for the original or similar research purposes compared with foregoing the study altogether.

As a reflection, if the proposed low-intervention study were to be developed in tandem with a nonfunded study, we consider that, obviously, the benefits of funding and use of resources would be lost. Therefore, when considering the possibility of such studies, one of the main criteria is likely to be that the context or the study on which it is based is funded or standard practice, as is the example of the study proposed here.

It may not be so much a question of whether to obtain funding but rather of making existing funding more efficient and taking advantage of the sometimes scarce resources available for research. We believe that rigorous, relevant, pertinent, and original research can be carried out, even without funding, if creative solutions are devised, such as the one that this article aimed to provide: taking advantage of existing resources to carry out low-intervention but rigorous studies from an experimental point of view. In this sense, would higher quality research be possible if funding was available? It seems obvious that the answer is yes but not in the sense of being rigorous or methodologically robust (which is mandatory when it comes to research). Rather, the answer is related to the sense of opportunity, of deciding what I want to investigate, and when and where I am going to do it. For the rest, we believe that it is even an obligation in the use of resources.

Most of the literature consists of partnership sponsors [10], budgeting [1,5], qualifications of research staff [31], or data contributions from registries [34]. However, a description of a nonfunded, low-interventional clinical trial and its corresponding results was identified for analysis in future research [8]; thus, no comparison is possible in the current article. Therefore, we suggest that our management description can be used for other researchers to conduct a clinical trial without funding or with limited funding.

### Strengths and Barriers of the Field and Study Context

According to Rogers’ theory, the adoption process depends on the characteristics of actors (such as values, skills, status), situational perception (such as norms, economical aspects), motivation, and information [16]. Thus, adoption depends not only on the individual position, which is conditioned by the collective one, but also on other environmental factors. In our specific noneconomic health care environment, we found strengths and barriers to change.

Regarding strengths, of all the nurses in the hospital, the nurses (variable staff) working in the unit were the most aware of research and innovation. However, the fixed research staff were aware and accepted that additional things were conducted, but they did not collaborate actively; they were only observers.

Regarding barriers, we cannot forget that the nursing profession is one of the least sensitive to innovation and only collaborates with economic incentives. Moreover, the fact that it is a variable component makes constancy difficult, both in the introduction of a new procedure and in the acceptance of changes. Although they were offered recognition in publications or similar, the big barrier was the lack of a direct financial incentive.

### Limitations

Our case is not applicable to all commercial trial designs, but it enables the creation of an option of management that can be adjusted according to the study field and commercial trial. Although our strategy could not guarantee the optimum design for ambitious aims, the proposed strategy could make it possible to conduct a nearly optimal study design and, therefore, provide results for research progress.

This article provides a strategy for conducting noncommercial or nonfunded clinical trials by including similar procedures in a funded study in order to reduce budget, personnel, and the cost of providing participants with extra conveniences.

Consequently, another limitation could be that knowledge about research methodology along with change theories or strategies is required. In this study, there was a research nurse with knowledge about change theories for innovation; therefore, we suggest this innovative management to help anyone who has to face a similar challenge.

### Conclusion

Our strategy is a cost-effective means of conducting a low-interventional clinical trial with no funding or with limited funding. Furthermore, this strategy can be used by nurse researchers or other researchers to facilitate clinical trial design and site management to provide high-quality results without ethical concerns. Ideally, nurses engaged in care themselves should be able to pose research questions like research nurses, develop them as such, and not be figures with necessarily distinct roles.

## Abbreviations

CONSORT: Consolidated Standards of Reporting Trials
WHO: World Health Organization

## Data Availability

The data sets generated and analyzed during this study are not publicly available but are available from the corresponding author on reasonable request.
